# Ranking the Effects of Urban Development Projects on Social Determinants of Health: Health Impact Assessment

**DOI:** 10.5539/gjhs.v6n5p183

**Published:** 2014-05-30

**Authors:** Parisa Shojaei, Masoud Karimlou, Jafar Nouri, Farahnaz Mohammadi, Hosein Malek Afzali, Ameneh Setareh Forouzan

**Affiliations:** 1 Social Determinants of Health Research Center, University of Social Welfare and Rehabilitation Sciences, Tehran, Iran; 2 The School of Public Health, Tehran University of Medical Sciences, Tehran, Iran

**Keywords:** Analytic Hierarchy Process, social determinants of health, man-made lake, health impact assessment

## Abstract

**Background and Objective:**

Health impact assessment (HIA) offer a very logical and interesting approach for those aiming to integrate health issues into planning processes. With a lot of works and plans waiting to be done (e.g., developing and updating plans, counseling planning commissions, cooperation with other organizations), planners find it difficult to prioritize health among a variety of possible issues and solutions they confront.

**Method:**

In the present article, first, the list of social determinants of health associated with Chitgar man-made lake was extracted out using a qualitative method and with content analysis approach, and then they were prioritized using analytic hierarchy process.

**Results:**

28 social determinants of health including “intermediary” and “structural” determinants were extracted out. Regarding positive effects of lake on these determinants, “recreational services” and “traffic” received the highest and the lowest weights with 0.895 and 0.638 respectively among structural determinants and with consideration to “construction” option. Furthermore, among intermediary determinants for “construction” option, sub-criteria of both “physical activity” and “air quality” received the final highest weight (0.889) and “pathogenesis” indicated the lowest weight with 0.617. Moreover, lake demonstrated the highest negative effects on “housing” among “structural” determinants which it takes the highest weight (0.476) in “non-construction” option. Additionally, lake had the highest negative effects on “noise pollution” among “intermediary determinants” and it takes the highest weight (0.467) in “non-construction” option.

**Conclusion:**

It has been shown that urban development projects such as green spaces, man-made lakes … have a huge range of effects on community’s health, and having not considered these effects by urban planners and mangers is going to confront urban health with many challenges.

## 1. Introduction

In most of the cases, urban planning entities are not the only elements or even, particularly, the main responsible institutions for health-related goals in the urban planning process, but the cooperation between these entities with planning process is quite necessary. There are some other social, economic, and environmental organizations involved in this process. For such conditions, we need a cooperative approach, i.e. a common activity towards reaching pre-agreed goals. This cooperation in urban planning gets both approaches together in order to have consciously decisions and efforts for society interference. Some part of this process requires identifying interest groups in the society who owns the benefits of adversaries in the decisions (Barati, 2011). The meeting point between health and urban planning has engrossed much interest so far. Practitioners are looking for clear-cut, systematic, and wide-ranging means to incorporate health concerns into planning processes. There is a substantial discussion about such matters, and a wide range of literature points to associations between the two disciplines. While the practice is rhetorically long, it is unluckily short when it comes to execution. However, perfectly and entirely accounting for health in plans and policies remains a key challenge. Health, however, is a vital issue in urban areas, and as the world goes towards urbanization, the link between cities and health will become gradually more important. HIAs are one means to help these challenges out, providing a rubric to bring health into planning. They can assist planners to systematically consider a variety of health concerns, serving as a tool to evaluate plans, policies, and development proposals. However, considering the current status of HIAs, there is still substantial uncertainty about how or when they should be used and what they say in the end. In many cases, the applicability of planning does not immediately become obvious. The urban planning community requires clear-cut yet comprehensive tools that help planners go forward through this cause in a way that is also nonthreatening and easy to utilize (“Design for Health. Building public understanding: The link between health and planning,” 2007). An HIA is a systematic appraisal that amalgams scientific data, professional proficiency, and stakeholder participation to find out the effects that a potential policy, plan, program, or project might have on the health of a particular population. HIAs offer information to decision-makers that can help minimize the expected adverse health effects and maximize positive health outcomes. It can be used for a variety number of sectors and can be employed at the federal, state, tribal, and local levels (“National Research Council of the National Academies (NRCNA). Improving Health in the United States: The Role of Health Impact Assessment.”, 2011). There exists a wide range of studies about evaluation of health effects of urban development projects which most of them are about green spaces such as parks, and a few have particularly focused on urban man-made lakes. As defined by Green Space Scotland (2008), the term “green space” can be applied to any vegetated land or surface water body within or adjacent to an urban area, including natural and semi-natural habitats; countryside immediately adjoining a town which people can access; green corridors—paths, rivers and canals; amenity grassland, parks and gardens; outdoor sports facilities, and playing fields (Greenspace Scotland, 2008). Therefore, the man-made lakes are green space and thus have the effects of a green space on heath. In a study carried out by Santana et al namely “evaluation of health effects of walkable urban green spaces”, they stated that these spaces have both direct and indirect impacts on health, and not only are connected with good health condition of habitats but also with environmental quality improvement(Santana, Santos, & Costa, 2009)Results of using HIA in urban planning process of Manukau city in Tailand indicated that it reinforces the intersection, especially structural one, between city council, interested people, and organizations out of the council which we do not see much of such cooperation at the time of urban planning (Field, September 2011). Therefore, considering health issues in urban plans and projects is really important. Creating man-made lakes in developing countries is followed by a lot of dangers. Stakeholders need to actively integrate in face-to-face cooperation in order to fully utilize, improve, and maintain water sources and lakes projects. As it looks, it is essential to have health professionals especially biologists, sociologists, and economists during the planning and implementation processes of development projects besides engineers (Araoye, 2002). Constructing the biggest man-made lake in north west of Tehran in district 22 is one of these under-construction projects in Iran. Chitgar man made lake, located in the urban District-22 Municipality of Tehran northwest of Tehran, Iran is by volume l0 Million Cubic Meter (MCM), by depth average 10 meters (m.) and by area 225 hectares (ha) as the Iran’s largest man-made lake. Approximately 80% of its water is supplied by Can river and the rest by run-off of Intermediate and surface area of the region (Nouri, Rahimipour, & Nezakati, 2004). History of construction the lake project goes back to develop the first comprehensive plan of Tehran city in 1986 which anticipated construction a lake in west of Tehran. But construction of the lake due to technical restrictions and budget remain dormant for years. Finally, in the background studies, since 1382-1389, by the consultant detailed studies were done at different times and uncertainties survey was completed. on October 2010, Catchment area of operations and on July 2012 coastal zones operations were began by the supervision of Sabir Company according to study and design of Tunnel Pars engineering company (Eshtuky Pars Company). The first phase of the project opened named Martyrs Persian Gulf on May 15th 2013 (Chitgar man made lake, 2013). It includes three Islands as well as green, recreational, playing and sport spaces. Although the objective of constructing man-made lakes is to create areas for attracting tourists, improve recreational capacity of area, and bring people joy and happiness morale, not considering environmental and health issues in designing, implementation, and utilization of such areas can have negative environmental and health consequences. With consideration to what mentioned above and a need to evaluate health effects of urban development projects, a study namely “Health impact assessment of constructing man-made lake in city development strategy on health and social determinants of health” is under implementation. The current article is taken from the “identification” step of above study which aims to identify and prioritize social determinants of health in the existing study. Based on the health impact assessment guidelines, multi-criteria decision-making model was used as a quantitative method in the present article (Harris, Harris-Roxas, Harris, & Kemp, 2007). Additionally, the Analytic Hierarchy Process (AHP) is a structured method for organizing and analyzing complex decisions, based on mathematics and psychology. It was developed by Thomas L. Saaty in the 1970s and has been widely studied and developed since then. AHP provides the treatment for complicated problems with multiple criteria, stakeholders, and decision makers in a scenario with a high uncertainty and high risk. It offers a good compromise between the target, understanding, and objectivity to the extent that is a tool supported by the basic mathematics letting ordinary people arrange tangible and intangible factors in a process of conflict resolution or order of priorities (Parra-Lopez, Calatrava-Requena, & de-Haro-Gimenez, 2007). Despite the employment of specialists for the realization of judgments, the AHP provides the likelihood that such judgments may remain inconsistent. In this sense, the AHP allows to assess the consistency and to find out the degree of inconsistency in a matrix of parity judgment (Thomas L Saaty, 2005). At this point, it is significant to consider that AHP is a technique for selecting the best alternative that integrates qualitative concerns and quantitative factors to the subjective process of decision-making, i.e., it lets the decision maker deal with the intuitive, rational, and irrational aspects simultaneously. This model has four stages ranging from creation of the decision problem, measurement and data collection, establishment of standard weights to presentation of summaries of findings solutions to the problem (Rosa & Haddad, 2013).

## 2. Methods

This study was two step that step A is Qualitative method for Extracting social determinants associated with artificial lakes and step B was weighting and prioritizing of its determinants.

A-Qualitative Method:

This qualitative study was carried out with a content analysis approach. Data were collected by individual interviews as well as focus group discussions (FGDs) from September to March 2012 respectively. Individual interviews were done on 30 informants in three groups: 1- people living in the 22 District, 2-Members involved in the man made lake project from the Municipal District 22 with following characteristics: educated individuals with at least B.A degree, experts on urban development, water sources, environment, sociology and urban planning, civil engineering; managers and administrative-executive experts in region 22 of municipality of Tehran, experts of Civil engineering consulting firms with at least one-year work experience, lake surroundings residents, and local authorities (managers of local health houses) and 3-third group to avoid information bias include Members from outside the municipal District 22 and experts in the field of health determinants and environment with at least a bachelor’s degree. The focus group discussions were carried out with 2 experts group that participated in individual interviews. Sampling was conducted using purposive in this study and until data saturation and new ideas and insights was driven. The focus group discussion have ability to explore people’s ideas, worries, attitudes and experiences of individuals regarding a specific subject matter (Barbour & Kitzinger, 1998; Dahlgren, Emmelin, & Winkvist, 2007). Individual interviews lasted for 50 minutes. For implementation of Interviews were used of focus group discussion guiding questionnaires and in-depth individual interviews. These procedures were designed based on the research team’s perspectives and experts who were familiar with health and urban development issues as well as library sources and objectives of the study. The concepts discussed in this questionnaire includes various health dimensions of man made lake construction. Validity of questionnaires was conducted through a pilot study, and necessary modifications in content, sequence and timing done. We arranged sessions with municipality coordination to make them aware of the study and asked for any permission required. In addition, at the beginning of interview informants were justified about the whole research, from its objectives to the methods and confidentiality of the data, and all agreed to take part voluntarily. All interviews were guided by the principal investigator. Focus Group discussion was held with the coordinating investigator for one and a half hours on Location with Participants agreed in the office of the honourable Deputy Mayor of Tehran 22. In meetings of individual interviews and focus groups were used of public and comprehensive questions and it progressed to detailed questions by the time. Interviews and group discussions were recorded and verbatim was rewritten. We used Graneheim approach for data analysis (Graneheim & Lundman, 2004). Therefore rewritten texts several times reread and meaning units was identified, and were coded finally.

Next, codes based on the meaning units represented by informants and their similarities and discrepancies were extracted and grouped, then from these groupings, themes were identified. At the beginning of the data analysis, according to the list of common social determinants of and core categories, data analysis was conducted using content analysis matrix (Averill, 2002). Then, with analysis continued, new categories were extracted as and they were put in the body of the total data analysis. In order to increase reliability all codes and themes were checked mutually by research team, and its summary was given to the informants in the end. The objectivity of the data, coded by two researchers were also and codes and categories were compared together. To trustworthiness of findings, data were collected based on the age, gender, and education of each informant separately. Additionally, other methods for assuring data trustworthiness include: Combining the methods of data collection and individual interviews as well as FGDs with each other, long-standing relationship with participants and research topic, extensive and detailed research report writing, reviews by participants and observers. In order to uphold the ethics of research, these points were met: Written and informed consent, permission to collect and record the data, maintaining the anonymity of the participants, the right to withdraw from the study, send results to all stakeholders and obtaining approval of the ethics committee.

B: AHP and Matrix Construction

**Figure 1:**
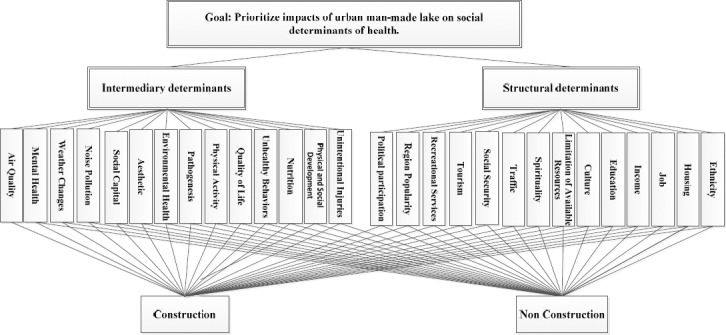
Different criteria and sub-criteria under investigation and their hierarchy

In this step, after analyzing content analysis methodology by the help of OpenCode software, list of all health determinants associated with man-made lake was extracted out. AHP is a structured method for organizing and analyzing complex decisions, based on mathematics and psychology. It was developed by Thomas L. Saaty in the 1970s (T.L Saaty, 1980). Its indispensable feature is that it changes individual preferences into ratio scale weights so providing the opportunity for effective comparison and ranking of the decision aspects. It has been utilized in various areas ranging from key factors of urbanization (Thapa & Murayama, 2010), fire risk analysis, engineering decision making, project selection, evaluation and management, vendor selection, housing sector, banking to marketing. The key features of AHP include hierarchical structure of complexity, pair-wise comparisons, unneeded judgments, an eigenvector method for providing weights and steadiness considerations (Bhatta & Doppler, 2010). As owned by many other methods, AHP provides opportunities for decision makers to build a model of a complicated problem with the goal at the top and criteria, sub-criteria (factors) and alternatives at levels in drop-down manner(Thomas L Saaty, 2008). Figure 1 shows the general framework of AHP used in the study. Since seven criteria were chosen in order to find their impact on the predominance of a particular farm practice, the dimension of the matrix, therefore, is 7×7. In this sense, one column and one row correspond to each factor and there were 21 pair-wise comparisons using the formula {n*(n-1)/2}. If this matrix is denoted as A={aij} where aij is the element of ith row and jth column of the matrix, all it’s entries are obtained by marking the relative importance of each criterion over another with consideration to the goal. Pair-wise comparison can be done by adopting an integer ranging from 1 to 9 or the mutual of such an integer to each cell of the matrix to gauge the relative importance of the factors that characterize the cell (T. L. Saaty, 1980). According to this scale, the available values for the pair-wise comparisons are members of the set: {9, 8, 7, 6, 5, 4, 3, 2, 1, 1/2, 1/3, 1/4, 1/5, 1/6, 1/7, 1/8, 1/9} (see also Table 1). According to the AHP approach assumption, each of the factors under assessment is independent of another. It makes some small variation in the judgment since human responses are not always constant. In practice, we can hardly achieve 100% consistency, but the method can still be employed when there is some degree of interdependence. In order to calculate the index of inconsistency, AHP makes use of consistency ratio (CR). Values of inconsistency index lower than 10% are acceptable, especially if matrix is 4 by 4 or above. In some cases, although higher value of inconsistency index asks for re-evaluation of pair-wise comparisons, decisions acquired in such circumstances could also be taken as ‘the best alternative’. Consistency index (CI) is calculated as (Yu, Wang, & Gong, 2013):

**Table 1:** Scale of relative importances (according to Saaty (1980))

Intensity of Importance	1	3	5	7	9	2,4,6,8
Definition	Equal importance	Weak importance of one over another	Essential or strong importance	Demonstrated importance	Absolute importance	Intermediate values between the two adjacent judgments


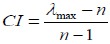


At the beginning, a hierarchical tree with its aim, criterion, sub-criterion, and options was formed for executing hierarchical analysis. Then all determinants extracted from qualitative method were put in a checklist in the form of pair-wise comparisons matrix in order to weigh them. In the next step, weights were assigned to each of them by the same participants participated in interviews (experts of second group and third group) using Saaty method. In order to facilitate the completion of this checklist by participants, it was completed once by having a positive approach on the effect of lake on determinants and again with consideration to its negative effects on health determinants. In this way, it might be possible to understand that which determinant is affected the most by the positive effects and the negative effects of lake construction. Indeed, everyone completed both checklists thereby helping us to have their both negative and positive points of view.

After having checklists completed, comparative tables of each respondent were combined together using geometric mean. Since pair-wise scales create data in the form of ratio, therefore, geometric mean seemed suitable among other methods. Since the interview subjects that completed this matrix were two groups of experts, so after completing paired matrix, a geometric mean was calculated for each group. Then the average of the two groups were evaluated by a panel of experts on health determinants. Finally, the review panel was formed matrix showed that the overall mean And the matrix for analysis and measurement of weights as raw data was used into the Expert choice software.

## 3. Results

This study was conducted in district 22 of Tehran municipality in 2012. Mean age of participants was 30, and they were experts in urban development, environmental, water source, civil engineering, sociology, urbanization, and health determinants areas. Their education ranged from B.S to P.H.D, and four of them were indigenous to the region and the others were from other parts of the city.

Qualitative analysis of data provided us with 261 codes. Furthermore, conceptual framework structure developed by commission on social determinants of health along with their sub-groups was considered as data analysis matrix (O. Solar & A. Irwin, 2010). Thus, two “structural” and “intermediary” groups were formed, and 14 sub-groups were determined in each stratum. Then these 28 determinants were put in a checklist and were given to participants through focus group discussion and interviews in order to implement AHP process. Therefore, we had one goal, two criteria, and 28 sub-criteria as well as two options (construction and non-construction of man-made lake) for depicting their hierarchy.

Findings regarding positive effects of lake construction on social determinants of health:

According to the findings, the maximum relative weight among structural determinants was calculated for income and tourism.

**Figure:**
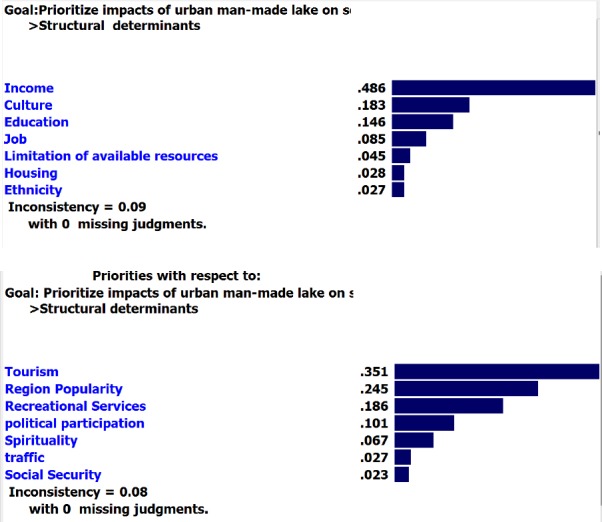


Moreover, “air quality” and “physical and social development of children’ had the highest weight among intermediary determinants.

**Figure:**
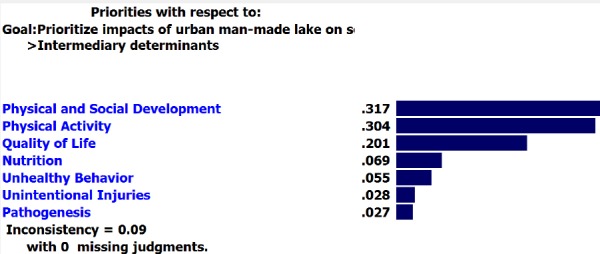


**Figure:**
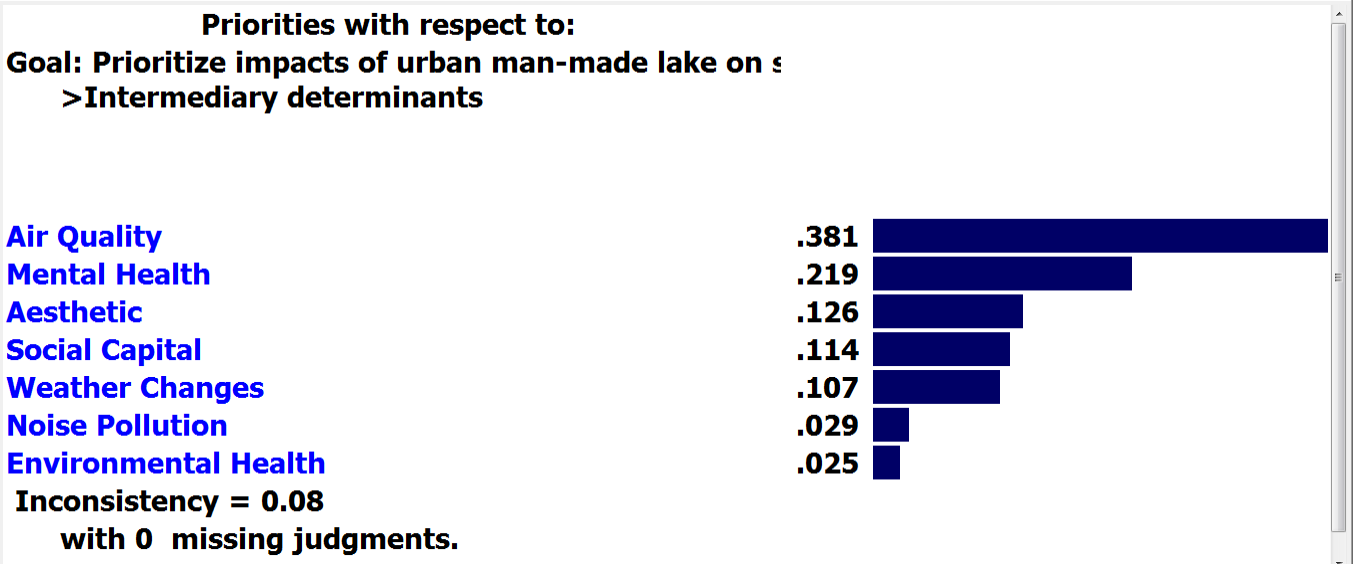


As seen in Table 2, regarding “construction” option, “recreational services” and “traffic” received the highest and the lowest weights with 0.895 and 0.638 respectively. These criteria, for sure, had the lowest and the highest weight for “non-construction” option with 0.105 and 0.362 respectively.

**Table 2:** Weightings of assessment criteria and sub-criteria in positive impact

Criterion/Weight	Sub criteria	Options Final Weight	

		Construction	Non Construction
**Structural**	Income	0.889	0.111
**Determinants**	Culture	0.857	0.143
Construction: 0.636	Education	0.873	0.127
	Job	0.833	0.167
Non Construction: 0.364	Limitation of available resources	0.750	0.250
	Traffic	0.638*	0.362*
	Ethnicity	0.804	0.196
	Tourism	0.889	0.111
	Region Popularity	0.891	0.109
	Recreational Services	0.895*	0.105*
	political participation	0.863	0.132
	Spirituality	0.860	0.140
	Housing	0.667	0.333
	Social Security	0.681	0.319
**Intermediary determinants**	Physical and Social Development of Children	0.877	0.123
Construction: 0.602	Physical Activity	0.889*	0.111*
	Quality of Life	0.857	0.143
Non Construction: 0.398	Nutrition	0.783	0.217
	Unhealthy Behavior	0.750	0.250
	Unintentional Injuries	0.786	0.214
	Pathogenesis	0.617*	0.333
	Air Quality	0.889*	0.111*
	Mental Health	0.869	0.131
	Aesthetic	0.857	0.143
	Social Capital	0.872	0.128
	Weather Changes	0.836	0.164
	Noise Pollution	0.667	0.333
	Environmental Health	0.625	0.375

About intermediary determinants for “construction” option, sub-criteria of both “physical activity” and “air quality” received the final highest weight (0.889) and sub-criterion of “pathogenesis” indicated the lowest weight with (0.617). For “non-construction” option, the above sub-criteria had the lowest and the highest final weights with (0.111) and (0.375) respectively.

Findings regarding negative effects of lake construction on social determinants of health:

Findings indicated that, among structural determinants, “traffic” and “housing” had higher weight compared to other elements.

**Figure:**
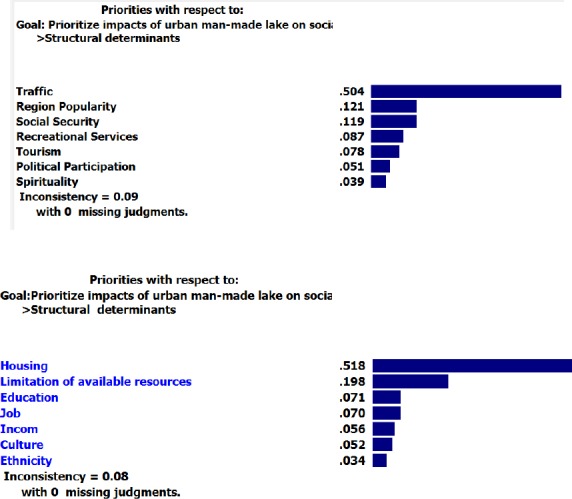


Moreover, “air pollution” and “pathogenesis” received relatively the highest weights among intermediary determinants.

**Figure:**
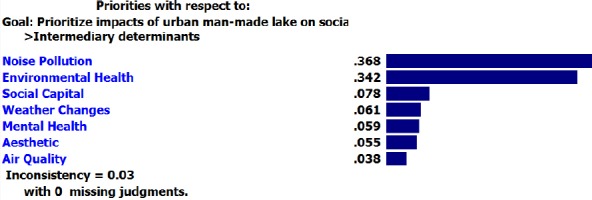


**Figure:**
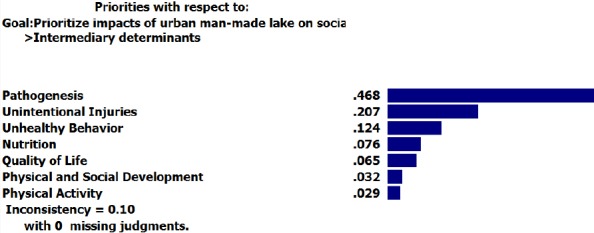


Final weights of criteria and sun-criteria for two “construction” and “non-construction” options are shown in table 3 with regard to their negative effects. As stated, lake has the highest negative effects on “housing” among structural determinants which it takes the highest weight (0.476) in “non-construction” option.

**Table 3:** Weightings of assessment criteria and sub-criteria in negative impact

Criterion/Weight	Sub criteria	Options Final Weight	

		Construction	Non Construction
**Structural determinants**	Income	0.889	0.111
Construction: 0.856	Culture	0.813	0.187
	Education	0.833	0.167
Non Construction: 0.135	Job	0.817	0.183
	Limitation of available resources	0.609	0.391
	Traffic	0.573	0.427
	Ethnicity	0.805	0.195
	Tourism	0.896	0.104
	Region Popularity	0.889	0.111
	Recreational Services	0.895	0.105
	political participation	0.834	0.166
	Spirituality	0.811	0.189
	Housing	0.524	0.476*
	Social Security	0.667	0.333
**Intermediary determinants**	Physical and Social Development of Children	0.875	0.125
Construction: 0.856	Physical Activity	0.890	0.110
	Quality of Life	0.875	0.125
Non Construction: 0.135	Nutrition	0.676	0.324
	Unhealthy Behavior	0.571	0.429
	Unintentional Injuries	0.643	0.357
	Pathogenesis	0.539	0.461
	Air Quality	0.900	0.100
	Mental Health	0.892	0.108
	Aesthetic	0.857	0.143
	Social Capital	0.860	0.140
	Weather Changes	0.820	0.180
	Environmental Health	0.564	0.436
	Noise Pollution	0.533	0.467*

Additionally, it has the highest negative effects on “noise pollution” among intermediary determinants and it takes the highest weight (0.467) in “non-construction” option.

## 4. Discussion and Conclusion

As broadcast in the findings, “recreational services” and “traffic” demonstrated the highest and the lowest final weights with regard to positive effects of lake on social determinants of health. It means that with consideration to sub-criterion of “recreational services,” “construction” option should be selected. Furthermore, as participants declared their points in qualitative study, Chitgar man-made lake and green spaces surrounding it provide a wide range of recreational facilities for people living there. King County’s Open Space System (2004) specified that everyone enjoy recreational activities. Parks, recreational and open spaces “provide places to exercise, take part in competitive sports, socialize with others, and a space for people to stand far from development and experience the natural environment,” which all play roles in physical, mental, and emotional health of people. Recreation paves the ways for us to learn, explore, and challenge things that contribute to human growth. Moreover, cultural and historic places play roles in shaping our sense of community identity and relationship with others (King County’s Open Space System: Parks, Trails, Natural Areas, and Working Resource Lands, 2004). A community’s park system can offer passive and active recreational chances close to home for a variety of residents and tourists. Neighborhood and community parks meet urgent urban area needs (Enger, 2005). Urban regeneration is defined as creating sustainable places, such as green spaces. These places should offer high-quality recreational services for urban residents (Arnberger, 2012). With consideration to “traffic” sub-criterion, “non-construction” option would be the priority. According to the participants, lake construction brings in a lot of visitors and tourists and will increase the traffic in the region which will have negative impacts on people’s health. Findings of researches done by South Carolina Institute of Medicine and Public Health (2013) demonstrated that the potential creation of a park could bring in visitors from outside the community, which could raise the traffic and reduce safety for pedestrians and cyclists. Crashes between motor vehicles and pedestrians or cyclists can cause injury or death. These incidents of injuries happen more often in areas with large amounts of pedestrians and motor vehicles, and children and elderly are most likely the victims of the related accidents (South Carolina Institute of Medicine and Public Health (IMPH). A Health Impact Assessment (HIA) of Park, Trail, and Green Space Planning in the West Side of Greenville, South Carolina, 2013). In addition, from the aspect of positive effects of lake on intermediary determinants, findings indicated that “physical activity” and “quality of air” received the highest weights among these elements. The lake and surrounding green spaces with recreational areas and sports fields - for hiking, rafting, … provides the opportunity for individuals to exercise, do physical activity and lessens obesity and its related diseases. Findings of an urban heart study in 2012 in Tehran showed that the percentage of overweight and obese men and women was high in one of the neighborhoods near the lake in District 22 of Tehran which the lake might have effects on decreasing those. Adjustment of the built environment makes green spaces augment chances for advantageous ‘green exercise’ such as walking (J. Pretty, M. Griffin, M. Sellens et al., 2003). According to Godbey et al., there exists possible spare-time physical activity in a variety of community environments such as local parks (Godbey & AJ, 2003). Several studies go with this attitude that ‘the built environment can facilitate or constrain physical activity (Bedimo-Rung, Mowen, & Cohen, 2005)’. In a European study carried out in eight cities, high levels of greenery caused people to be physically active three times more than the others and the chance of being overweight and obese was about 40% lower for these people than for those living in similar areas with lower levels of greenery. On the other hand, people living in areas with high levels of anti-social activities were less likely to be physically active and more likely to be overweight or obese (Ellaway, Macintyre, & Bonnefoy, 2005). In a systematic review of 50 quantitative studies investigating the associations between green space access and physical activity, 20 reported positive links (higher physical activity with increased green space access), 15 were weak or mixed, 2 were negative and 13 found no evidence of any association(Lachowycz, Jones, Page, Wheeler, & Cooper, 2012). Result of present study indicated that Chitgar man-made lake with its surrounding green spaces as new ecological zones helped to soften the air in the area. Since the particular heat capacity of water is high, the lake water can accumulate large amounts of heat. This sometimes causes a decline in the magnitude of temperature alterations. South Carolina Institute of Medicine and Public Health (IMPH) assessed the health effects of park, forest paths, and green spaces plans in west part of Greenville, and found out “quality of air” as one of the important determinants connected to these spaces (South Carolina Institute of Medicine and Public Health (IMPH). A Health Impact Assessment (HIA) of Park, Trail, and Green Space Planning in the West Side of Greenville, South Carolina, 2013). Moreover, Faculty of Public Health of London found improvement of welfare and mental health for all, better physical activity, declined violence, and anti-social behaviors, reduced health disparities, reduced mortality and cardiovascular diseases, enhancement of air quality, solving noise pollution, and fiscal profits as health determinants connected to green spaces (Faculty of Public Health. Great Outdoors: How Our Natural Health Service Uses Green Space To Improve Wellbeing, 2010). According to the Heinze, healthy and appropriately maintained green space offers indispensable benefits to the environment in terms of water refinement, air refinement, and temperature change (Heinze, 2011). “Non-construction” option was also chosen with regard to sub-criterion of pathogenesis. Hunter et al. pointed out that construction of man-made lakes and dams as well as development of irrigation projects in tropical areas are especially implicated because, besides the obvious profits brought to the economy of a country, they have a tendency to ruin the environment by devastation of forests, increasing soil erosion, and the production of more biotypes than before to intermediate hosts or vectors of parasitic or infectious diseases. Such parasitic and infectious diseases transmission cycles profoundly affect series of ecosystem (Hunter, Rey, & Scott, 1983).

A policy position is recommended whereby disease prevention procedures are incorporated with development projects from the scratch along with identification and inclusion of infrastructural investment and operational costs for health maintenance in the total benefit-cost analysis (Hunter, Rey, & Scott, 1982). “Noise pollution” came to have the highest weight with regard to negative effects of lake on intermediary determinants. Potchter stated that urban areas suffer from a variety of environmental troubles because of human activities which boost the development of urban heat islands, augment air pollution and noise. This study examines the potential of vegetation to improve local air quality, mitigate air temperature, and reduce noise levels. Also, in a study carried out in the city of Tel Aviv, Israel, they investigated the total environmental consequences of diverse green urban spaces on climate, noise and air pollution (Potchter, Shashua-Bar, Choen, Boltansky, & Yaakov, 2009). Most European cities are now facing with major socioeconomic problems such as interrelated issues of urban sprawl, traffic congestion, noise, and air pollution. A methodology is now being introduced for assessing the role of green space and urban form in mitigating the adverse effects of urban station, with a focus on the environment but also considering socioeconomic aspects. Of other negative effects of lake construction was on “housing”. Participants believed that lake construction raises the price of housing but it may decrease its quality. Such a rise in property values is considered as a potential power for more displacement and even homelessness because of high rents. The potential gentrification could also disable new people of alike economic conditions to come into the neighborhood. Furthermore, property taxes for homeowners may be increased as well. If they lack abilities to afford to stay in their homes, they could also be displaced. A rise in property values could result in the possibility for displacement due to gentrification. Gentrification points to the boost in property values as a result of renovation and redevelopment in poor areas (Huestis, 2005). This shift can have a significant influence on health differences, especially for the poor, women, and children, the elderly and racial minorities. Studies show that these vulnerable groups have proclivities for higher rates of asthma, diabetes, and cardiovascular disease. Those residents who are influenced by such shift can experience an alteration in stress levels, crime, and/or mental health. Other health related impacts of displacement can comprise a lack of access to healthy food choices, transport, and quality of schools, bicycle, and walking pathways as well as affordable housing (Center for Disease Control and Prevention (CDC), Health Effects of Gentrification, 2012). Klessig believed that sustainability should start from needs. What are the needs of community and which of them can be met by lakes are important, and through this way, they can play their roles in sustainability issues. He pointed out eleven needs essential for a community to consider. A sustainable social structure should provide measures to reach them, and so the functions of lakes can be defined accordingly as provision of aesthetic, cultural, economic, educational, and recreational opportunities as well as collective, emotional, environmental, spiritual, and individual safety (Klessig, 2001). Parks and urban green spaces as one of the most important public areas in modern cities play a very crucial role in meeting various social, cultural, and psychological needs of citizens. It is expected that with an increase in number of studies done on evaluation of health effects in Iran, the adverse effects of implementing projects, designs, and policies on people’s health would be recognized, prevented, and eradicated in coming futures.
